# A systematic review on the cost‐effectiveness of the computer‐assisted orthopedic system

**DOI:** 10.1002/hcs2.23

**Published:** 2022-11-02

**Authors:** Hua Li, Tengfeng Zhuang, Wenrui Wu, Wenyi Gan, Chongjie Wu, Sijun Peng, Songwei Huan, Ning Liu

**Affiliations:** ^1^ Department of Orthopaedics The First Affiliated Hospital of Jinan University Guangzhou Guangdong Province China

**Keywords:** computer‐assisted orthopedic system, cost‐effectiveness, systematic review and meta‐analysis

## Abstract

Computer‐assisted orthopedic system (CAOS) is rapidly gaining popularity in the field of precision medicine. However, the cost‐effectiveness of CAOS has not been well clarified. We performed this review to summarize and assess the cost‐effectiveness analyses (CEAs) with regard to CAOS. Publications on CEA in CAOS have been searched in PubMed and CEA Registry up to May 31, 2022. The Quality of Health Economic Studies (QHES) instrument was used to estimate the quality of studies. Relationships between qualities and potential factors were also examined. There were 15 eligible studies in the present review. Twelve studies evaluated CAOS joint arthroplasties and found that CAOS joint arthroplasties were cost‐effective compared to manual methods. Three studies focused on spinal surgery, two of which analyzed the cost‐effectiveness of CAOS for patients after spinal fusion, with conflicting results. One study demonstrated that CAOS was cost‐effective in spinal pedicle screw insertion. The mean QHES score of CEAs included was 86.1. The potential factors had no significant relationship with the quality of studies. Based on available studies, our review reflected that CAOS was cost‐effective in the field of joint arthroplasty. While in spinal surgery, the answer was unclear. Current CEAs represent high qualities, and more CEAs are required in the different disciplines of orthopedics where CAOS is employed.

## Graphical abstract

In the era of precision medicine, the computer‐assisted orthopedic system is fast growing. This review summarizes the cost‐effectiveness analyses with regard to computer‐assisted orthopedic system and explores the potential factors that would influence the cost‐efficiency of this new‐generation technique.

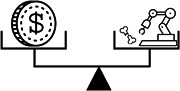

**Abbreviations**
CAOS: computer‐assisted orthopedic system
CEA: cost‐effectiveness analysis
CND: Canadian dollar
GBP: Great Britain pound
ICER: incremental cost‐effectiveness ratio
MIS: minimally invasive
NOK: Norwegian Kroner
PRISMA: the Preferred Reporting Items for Systematic reviews and Meta‐Analyses Statement protocol
QALY: quality‐adjusted life year
QHES: the Quality of Health Economic Studies instrument
RCT: randomized controlled trial
THA: total hip arthroplasty
TKA: total knee arthroplasty
UKA: unicompartmental knee arthroplasty
UK: the United Kingdom
US: the United States
WTP: willingness to pay

## 1 INTRODUCTION

Cost‐efficiency balancing is one of the areas of greatest concern in healthcare policy [1]. To achieve this, economic analysis is needed to assess the value of treatment and assist policymakers in allocating medical resources. Factors to be taken into consideration include the cost of treatment, as well as the quality and quantity of the patient's life. Cost‐effective analysis (CEA) is generally recommended as an important tool for the economic analysis of health care [2, 3, 4].

Computer‐assisted orthopedic systems (CAOS) such as robotics, computerized navigation and other computer‐assisted devices have burgeoned in all orthopedic subspecialties, especially in arthroplasty and spinal surgery [5, 6, 7], over the past two decades [8, 9, 10]. The use of CAOS offers surgeons convenient and precise operative manipulations [11, 12, 13, 14] and benefits patients with better clinical outcomes and lower failure rates of operations [15, 16, 17, 18, 19]. The development and application of CAOS are rapidly increasing [20, 21]. Given that the cost of CAOS is high, it is necessary to determine whether the accessional expenditures incurred by this new technique live up to the incremental outcomes as expected. Indeed, numerous studies have demonstrated the promising upsides of CAOS compared to traditional procedures [14, 22, 23]. However, recent studies on the efficacies of CAOS have shown controversial results. Bush et al. [24] found that an experienced surgeon could perform unicompartmental knee arthroplasty (UKA) by freehand even more precisely than using robotics. Passias et al. [25] reported the relatively higher costs of CAOS in lumbar fusion did not lead to a decrease in failure rate. Hickey et al. [26] pointed out that a myriad of comparative trials were underpowered to detect total knee arthroplasty (TKA) revision rate reductions due to CAOS. The authors subsequently conducted a simulated analysis. Although the precision of CAOS was set higher than that of the conventional method, the 15‐year survivorship of simulated TKA showed a difference of 2% in revision rate between the CAOS group and the conventional method group, and the sample sizes are required to include 5000 or more, which was barely practical for real randomized controlled trials (RCTs) [26]. In addition, results regarding CAOS should be cautiously interpreted because bias probably exists. A recent review of joint arthroplasty found that almost all studies (91%) comparing robotic‐assisted versus conventional techniques involved authors with financial conflicts, and studies with conflicts were more likely to report favorable outcomes of robotics than studies not associated with conflicts [27]. It is, therefore, arbitrary to state that CAOS is cost‐effective.

CEAs in different disciplines of orthopedics, such as traumatology, arthroplasty, spine surgery, and sports medicine [28, 29, 30, 31], have been fully discussed in previous systematic reviews; however, those in CAOS, the new‐generation devices, have been poorly studied. The purpose of this systematic review was to identify, summarize, and evaluate published economic analyses assessing the cost‐effectiveness of CAOS and to answer whether CAOS is cost‐effective.

## 2 MATERIALS AND METHODS

### 2.1 Background

In this paper, CEA refers to the four cost analyses of cost‐identification (minimization) analysis, cost‐effectiveness analysis, cost‐utility analysis, and cost‐benefit analysis [32].

The cost‐identification analysis identifies the option that achieves the same outcome at the lowest cost based on the cost of each treatment strategy. This form of analysis is thought to be easily accomplished, but is difficult to apply because the comparisons depend on a strict assumption that different interventions share equal outcomes and the cost is the only factor taken into account.

The cost‐effectiveness analysis focuses on the net cost divided by changes in health outcomes. The health outcomes should be objective parameters, such as prolonging life years, reducing revisions, or improving survival. Measuring the incremental cost and incremental health outcomes of an intervention allows the calculation of an incremental cost‐effectiveness ratio (ICER). A core aspect of CEAs is a decision maker's willingness to pay (WTP) to gain an extra health unit. The threshold of WTP is usually predefined. If the ICER is lower than the WTP, the intervention is deemed “cost‐effective” [33].

The cost‐utility analysis highlights the costs per health utility from the perspective of extending patients’ life spans and improving their health conditions. The health outcome measured in the cost‐utility analysis is a patient‐centric and subjective item, generally, quality‐adjusted life year (QALY), which represents both the quality and quantity of life. The QALY is a scale where 0 represents death and 1 represents full health. Lower QALYs represent the time spent with impaired physical and emotional function than the time spent in full health. The more severe the impairment is, the lower the value of QALY is [34]. The ICER could also be calculated by the incremental costs to gain an incremental QALY. Cost‐utility analysis is the preferred modality for reporting medical decision analysis [35].

The cost‐benefit analysis considers the costs of all input and output in monetary terms, upon which healthcare consumers are questioned whether they are willing to pay for health interventions that achieve a certain health outcome.

The evaluation of cost depends on the perspectives. Generally, the analytic perspectives can be categorized as follows: the payer perspective, which includes only the costs incurred in health service by a payer (typically a third party, such as Medicare); the healthcare system perspective, which includes all the costs of health service, regardless of who affords the costs; and the societal perspective, which includes the costs of health sectors, patients and productivity losses due to morbidity and mortality [36, 37]. The British National Health Service recommends using the payer's perspective to assist the government in making decisions about funding healthcare interventions, while the United States (US) Panel on Cost‐effectiveness in Health and Medicine encourages the use of the societal perspective to calculate indirect costs associated with interventions [28].

### 2.2 Search strategy and eligibility criteria

We conducted this systematic review following the Preferred Reporting Items for Systematic reviews and Meta‐Analyses (PRISMA) Statement protocol [38, 39] and retrieved literature up to May 31, 2022 from databases including PubMed and CEA Registry hosted by the Center for the Evaluation of Value and Risk in Health at Tufts University. The keywords in the title or abstract are listed as (“robotic” OR “navigate” OR “computer‐assisted”) AND (“cost‐effectiveness” OR “economic”).

Publications reporting the CEA for operations of CAOS were included. We excluded non‐English language reports, case reports, conference abstracts/posters, or reviews. As the cost‐identification (minimization) analysis requires a critical parity in outcomes among interventions, it is rarely applied correctly [32, 40, 41], so publications regarding this were also excluded. After the removal of duplicates, two reviewers independently reviewed the titles and abstracts to select potentially eligible studies. The full texts of them were then assessed independently by the same two reviewers to determine the final list of eligible publications for this study. In case of disagreement, a third senior reviewer was consulted for final assessment and consensus.

### 2.3 Data extraction

After the final list of included studies was set, data were extracted, including information on the publication, district, CAOS type, research design, perspective, time horizon, patients’ attribution, model, data resource, major findings, conflicts of interest, and funding. The primary outcome of interest was the outcome of the cost‐effectiveness evaluation.

### 2.4 Quality assessment

We used the Quality of Health Economic Studies (QHES) instrument, which is commonly used to assess the methodological quality of economic studies, to estimate the quality of the involved studies [42]. The QHES scores range from 0 to 100 and those above 85 are considered high quality [14].

### 2.5 Data synthesis

The publications were stratified according to the disciplines such as traumatology, spinal surgery, arthroplasty, bone tumor surgery, sports medicine, and so on. Given the heterogeneity of the reported forms of outcomes, descriptive analysis was used for collective data. Microsoft Excel 2016 (Microsoft) was employed to summarize the related information.

## 3 RESULTS

**Figure 1:**
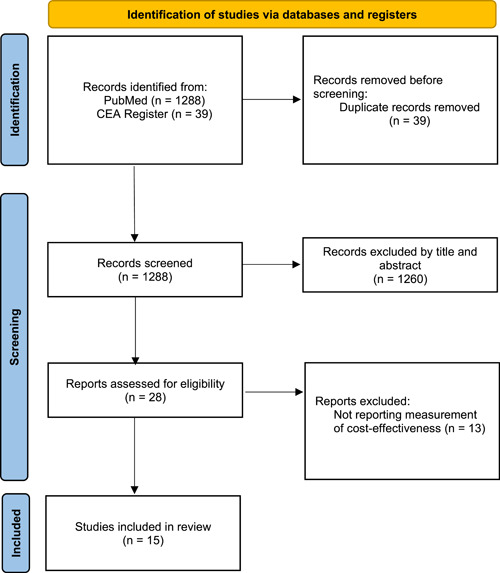
Preferred Reporting Items for Systematic reviews and the Protocol Flowchart of Meta‐Analyses Statement

There were 1288 studies found from the initial retrieval. After reading through the titles and abstracts, we excluded 1260 studies and reviewed the full texts of the remaining 28. Thirteen studies were then excluded and no other research meeting the inclusion criteria was identified in the reference lists. As a result, there were 15 eligible articles in this systematic review (Figure 1).

### 3.1 Overview of the included studies

The types of CAOS consisted of computerized navigation and robotics. Twelve studies focused on arthroplasty (six on TKA, four on UKA, and two on total hip arthroplasty [THA]) [43, 44, 45, 46, 47, 48, 49, 50, 51, 52, 53, 54], of which 11 used economic Markov models and 1 used retrospective data. Two studies focused on CAOS spine fusion (one using Monte Carlo simulation and one using a decision model) [25, 55]. One study used prospective data to investigate the cost‐effectiveness of CAOS pedicle screw insertion [56]. These studies were published between 2006 and 2022, 60% of which were published in the last 5 years. Of these 15 studies, nine studies were funded by the CAOS industry or conducted by authors with conflicts of interest. There were seven studies using the healthcare system's perspective, eight using the payer's perspective and none using the societal perspective. Eight and four studies were from the United States (US) and the United Kingdom (UK), respectively, and the rest came from Canada, Norway, and Belgium. The US dollar and the Great Britain pound (GBP) were the most commonly used currencies (Table 1).

**Table 1:** Overview of included research

Study	Year	District	Currency	Perspective	CAOS type	Area	Study design	QHES score
Yeroushalmi et al.	2020	US	USD	Payer	Robotics	UKA	Markov model	91
Clement et al.	2019	UK	GBP	Payer	Robotics	UKA	Markov model	88
Moschetti et al.	2016	US	USD	Payer	Robotics	UKA	Markov model	83
Nherera et al.	2020	UK	GBP	Payer	Robotics	UKA	Markov model	78
Rajan et al.	2022	US	USD	Payer	Robotics	TKA	Markov model	92
Vermue et al.	2021	Belgium	USD	Payer	Robotics	TKA	Markov model	92
Novak et al.	2007	US	USD	Healthcare system	Navigation	TKA	Markov model	92
Gøthesen et al.	2013	Norway	NOK	Healthcare system	Navigation	TKA	Markov model	84
Slover et al.	2008	US	USD	Healthcare system	Navigation	TKA	Markov model	82
Dong et al.	2006	UK	GBP	Healthcare system	Navigation	TKA	Markov model	70
Clement et al.	2022	UK	GBP	Payer	Robotics	THA	Retrospective design	88
Maldonado et al.	2021	US	USD	Payer	Robotics	THA	Markov model	92
Garcia et al.	2022	US	USD	Healthcare system	Robotics	Spine fusion	Retrospective design with a decision model	88
Passias et al.	2020	US	USD	Healthcare system	Robotics	Spine fusion	Retrospective design with Monte Carlo simulation	88
Dea et al.	2016	Canada	CND	Healthcare system	Navigation	Spinal pedicle screw insertion	Prospective design	84

Abbreviations: CAOS, computer‐assisted orthopedic system; CND, Canadian dollar; GBP, Great Britain pound; NOK, Norwegian Kroner; QHES, the quality of health economic studies instrument; THA, total hip arthroplasty; TKA, total knee arthroplasty; UK, the United Kingdom; UKA, unicompartmental knee arthroplasty; US, the United States; USD, US dollar.

### 3.2 Cost‐effectiveness findings

Table 2 summarized the major findings of each study.

**Table 2:** Major findings of included studies

Study	Time horizon	ICER/WTP threshold	Major findings
Yeroushalmi et al.	5 years	$50,000/revision avoided	The ICER of CAOS UKA was $7,271/revision avoided, $14,737/revision avoided and $28,716/revision avoided for ages <55, 65, and >75 years old, respectively. Beyond 7 years, CAOS became cost‐saving. Sensitivity analysis: Not sensitive to changes in revision probability, health utility of CAOS UKA, UKA robot cost, and discount rate.
Clement et al.	Life expectancy	£20,000/QALY	The ICER of CAOS UKA was £1395 per QALY and £1,170 per QALY relative to TKA and UKA, respectively. Sensitivity analysis: Sensitive to surgical volume and length of stay.
Moschetti et al.	5 years	$50,000/QALY	The ICER of CAOS UKA was $47,180 per QALY. Sensitivity analysis: Sensitive to surgical volume, CAOS UKA failure rate, discounted costs of the robotic system and maintenance, robotic system lifetime and patient's age.
Nherera et al.	5 years	£20,000/QALY	The ICER of CAOS UKA was £2,831 per QALY. Beyond 7 years CAOS became cost‐saving. Sensitivity analysis: Sensitive to surgical volume and patient's age; not sensitive to changes in revision rate, health utility of CAOS UKA, and discount rate.
Rajan et al.	Life expectancy	$50,000/QALY $100,000/QALY	The ICERs for CAOS TKA were $256,055/QALY (low volume, in institutes with a volume of 10 cases), $15,685/QALY (mid volume, in institutes with a volume of 100 cases), and $2,331/QALY (high volume, in institutes with a volume of 200 cases). Sensitivity analysis: Sensitive to QALY of CAOS TKA, CAOS UKA failure rate, and surgical volume.
Vermue et al.	20 years	$50,000/QALY	The ICER of CAOS TKA was $376,145 per QALY (>threshold) at 70 cases per year. At 253 cases, the CAOS became cost‐effective. Sensitivity analysis: Sensitive to surgical volume and the health utility of the primary and revision TKA.
Novak et al.	15 years	$50,000/QALY $100,000/QALY	The ICER of CAOS TKA $45,554 per QALY. The CAOS was cost‐effective with WTP of $50,000 and $100,000 beyond 14.56 and 12.13 years follow‐up, respectively. Sensitivity analysis: Sensitive to additional cost of CAOS, cost of revision, and probability of neutral alignment in manual TKA. Not sensitive to the probability of neutral alignment in CAOS TKA.
Gøthesen et al.	20 years	NOK 500,000/QALY	The ICER of CAOS TKA was NOK 1,037 and NOK 1414 per QALY, respectively, for 60 and 75 year olds under the surgical volume of 25 TKAs, and NOK 128 and NOK 175 per QALY, respectively, under the surgical volume of 250 TKAs. Sensitivity analysis: Sensitive to 10‐year implant survival and surgical volume.
Slover et al.	20 years	$50,000/QALY	The CAOS TKA could be cost‐effective with the requirement of relative reductions in the rate of revision for the following surgical volumes: 25/year, 13%; 150/year, 2.5%; and 250/year, 2%. Sensitivity analysis: Sensitive to surgical volume and an annual cost of CAOS.
Dong et al.	10 years	£30,000/QALY	The CAOS TKA reduced costs per patient by £583 and increased QALYS by 0.0148. Sensitivity analysis: Sensitive to the health utility of the primary TKA and added cost of CAOS.
Clement et al.	10 years and life expectancy	£20,000/QALY	The CAOS THA was associated with a greater improvement of 0.7935 QALYs in the 10‐year time horizon and 1.5470 QALYs in the lifetime horizon when compared to manual THA. The ICERs of CAOS THA were £1,910/QALY in a 10‐year time horizon and £980/QALY in a lifetime horizon. Sensitivity analysis: Surgical volume.
Maldonado et al.	5 years	$50,000/QALY	The CAOS THA reduced $945 for Medicare and $1,810 for private insurance with 0.04 QALYs gained.
			Sensitivity analysis: Sensitive to the health utility of the primary THA; not sensitive to the health utility of revision and cost of revision.
Garcia et al.	1 year	$50,000/QALY	The CAOS spine fusion cost $21,546.80 to gain 0.68 QALYs and manual spine fusion cost $22,398.98 to gain 0.67 QALYs. The ICER of CAOS spine fusion was ‐$70,562.50/QALY. Sensitivity analysis: Sensitive to operation room costs and procedure costs of CAOS.
Passias et al.	1 year and life expectancy	$100,000/QALY	The CAOS of spine fusion had the highest costs of $59,2734.30 after 1 year and $29,785.64 at life expectancy to gain a QALY. When comparing open surgery and MIS to CAOS, the ICER was $1,156 per QALY and $17,042 per QALY, respectively.
Dea et al.	4 years	NA	The ICER of CAOS for pedicle screw insertions was CND$15,961 per revision avoided and CAOS became cost‐saving at a surgical volume of over 254 procedures. Sensitivity analysis: Sensitive to the revision rate of CAOS.

Abbreviations: CAOS, computer‐assisted orthopedic system; CND, Canadian dollar; ICER, incremental cost‐effectiveness ratio; MIS, minimally invasive; NA, not applicable; NOK, Norwegian Kroner; QALY, quality‐adjusted life year; THA, total hip arthroplasty; TKA, total knee arthroplasty; UKA, unicompartmental knee arthroplasty; WTP, willingness to pay.

#### 3.2.1 Joint arthroplasty

Six studies compared COAS TKA with manual TKA in a time horizon of 10 years to lifetime expectancy [44, 45, 46, 50, 51, 53]. Only one study by Dong et al. [51] confirmed that CAOS had a dominant advantage, which meant that CAOS cost less and could help patients gain more QALYs. The authors also found that CAOS TKA had a lower rate of revision (5.35% vs. 7.74%) as compared with manual TKA in a 10‐year cohort simulation. Rajan et al. [53] also reported that CAOS TKA was able to achieve a lower rate of revision (0.6% vs. 1.5%) than manual TKA in a lifetime horizon. Five studies found that COAS TKA was conditionally cost‐effective [44, 45, 46, 50, 53]. Three studies determined that CAOS TKA could be cost‐effective with the accepted WTP threshold, but such performance could only be achieved when the surgical volume was high enough, at least over 250 CAOS cases per year [44, 45, 50]. In the study by Rajan et al. [53], a surgical volume of over 42 cases per year was needed for CAOS TKA to be cost‐effective. The revision rate and annual costs of CAOS, the health utility value after revision TKA and the WTP threshold also contributed to the uncertainty of cost‐effectiveness. Gøthesen et al. [50] found that CAOS TKA could be more cost‐effective in the younger patients.

Three studies compared the cost‐effectiveness of CAOS UKA with that of manual UKA over 5 years [43, 47, 48] and one study compared the cost‐effectiveness of CAOS UKA with that of manual UKA and manual TKA over life expectancy [52]. A WTP threshold of $50,000/QALY was used in the two US studies and £20,000/QALY in the two UK studies. The CAOS UKA was suggested to be potentially cost‐effective in all studies and even cost‐saving after a follow‐up beyond 7 years in two studies [43, 47]. Two studies reported that the employment of CAOS could lead to a reduction in revisions. Yeroushalmi et al. [43] reported that the application of CAOS could exempt 12 revision cases per 100 UKA cases. In the study by Nherera et al. [47], 10 revision cases per 100 UKA cases could be avoided. Among all the studies mentioned above, high surgical volume (over 100 CAOS UKA per year) was critical and emphasized and cost‐effectiveness was more favorable for the younger patients [48].

Two studies compared the cost‐effectiveness of robotic‐assisted THA with that of manual THA. Maldonado et al. [49] conducted a Markov model based on published data and found that CAOS THA was dominant over a 5‐year period. The health utility of primary CAOS THA or manual THA represented to be the only two sensitive elements that affected the cost‐effectiveness. Clement et al. [54] used retrospective follow‐up data of 560 patients (48 following CAOS THA and 512 following manual THA) from two centers, whose results revealed that CAOS THA was associated with an ICER of £1910/QALY in a 10‐year time horizon and £980/QALY in a lifetime horizon, both of which were below the given WTP threshold (£20,000/QALY).

#### 3.2.2 Spinal surgery

Garcia et al. [55] created a decision model to assess the cost‐effectiveness of robotic‐assisted minimally invasive (MIS) transforaminal lumbar interbody fusion based on institutional data from 76 patients. The researchers used CAOS to identify the location of the pedicle screws. Compared with manual MIS procedures, CAOS cost was lower ($21,546.80 vs. $22,398.98) and QALYs were higher (0.68 QALYs vs. 0.67 QALYs).

Passias et al. [25] also investigated the cost‐effectiveness of robotic‐assisted MIS lateral or transforaminal lumbar interbody fusion. In the Monte Carlo simulations, robotic‐assisted surgery was the most costly in the 1‐year or lifetime range based on institute data of 360 matched patients (120 open, 120 manual MIS, 120 robotic‐assisted MIS). However, the revision rates were comparable among the three groups (3% open, 3% manual MIS, 5% robotic‐assisted MIS, *p* > 0.05). Therefore, the robotic‐assisted MIS procedure was identified as dominant.

Dea et al. [56] reported their prospectively collected data on pedicle screw insertion in spinal surgery. The outcome of interest was the unplanned revision for symptomatic pedicle screw malposition. They found that revision rates for the computer‐navigated pedicle screw insertion were lower (0.8% vs. 6%) compared with traditional methods. The revision rate was reduced by 5.2% with the use of the navigation system. Assuming a cost per patient of CND$1123 for CAOS and CND$293 for the manual method, the ICER of CAOS was CND$15,961 per revision avoided. The navigation‐assisted strategy could be cost‐saving for a surgical volume of over 254 procedures. In addition, the ICER was sensitive to the revision rate.

### 3.3 Quality of CEA

**Figure 2:**
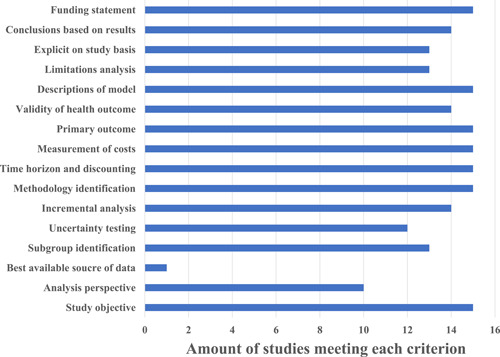
Results of Quality of Health Economic Studies criterion.

The overall mean QHES score was 86.1 (range: 70–92) and the mean quality scores for joint arthroplasty and spinal surgery were 86 and 86.7, respectively. Figure 2 showed the frequency with which the included studies met each QHES criterion. Study perspective (*p* = 0.112), industry‐funded or conflict (*p* = 0.411), and authors’ degree (more than 2 authors with a degree higher than MD) (*p* = 0.183) were not significantly predictive of obtaining a high‐quality CEA.

## 4 DISCUSSION

Our review summarized the CEA publication in CAOS and those studies were generally of high quality. Based on a paucity of available studies, the cost‐effectiveness of CAOS was certificated in the field of joint arthroplasty. In spinal surgery, however, the answer remains controversial.

To our knowledge, this is the first systematic review of CEA in CAOS. In the era of precision medicine, CAOS is developing rapidly, with the expectation of precise manipulation, better clinical outcomes, and longer survival [57]. However, recent studies question its additional effectiveness compared to traditional methods. Therefore, CEAs are appealed [24, 25]. This review reflected the increasing interest in CEAs in CAOS and the requirement for prudent evidence.

Our current systematic review highlighted the evidence of health utility clarified by CEAs and also estimated the qualities of analyses to find aspects where the methodological promotions of CEAs could achieve. There is only one previous publication that examined the cost‐effectiveness of CAOS in general, which reported four CEAs of navigation‐assisted TKA without quality assessments [58]. In particular, we noticed that none of the CEAs in the present review were conducted from the societal perspective recommended in the United States, as they included both direct and indirect costs [59].

In this review, there is no certain consensus but a sketchy range regarding the ultimate question of analyzing cost‐effectiveness, the WTP threshold. Studies using the US dollar system have generally used a threshold of $50,000–$100,000/QALY and those using the GBP system of £20,000–£30,000/QALY [60]. A procedure that is considered costly usually costs over $100,000 to gain a QALY. Nevertheless, researchers have questioned the scientific basis of the traditional value for this threshold, as it was determined several decades ago [61, 62]. In 2008, Braithwaite et al. [62] established a mathematical model based on insurance and mortality data and appealed that the acceptable lower and upper bounds for cost‐effectiveness were $109,000 per QALY and $297,000 per QALY, respectively. The World Health Organization recommended three times the gross domestic product per QALY [63], but the applied WTP threshold still varied within a limited range between previous values, even in the most recent publications. The WTP threshold is crucial for determining cost‐effectiveness and should be set prudently rather than crudely in future studies.

Previous CEAs and related reviews concerning manual joint arthroplasty have proved its cost‐effectiveness compared with no surgery [64, 65]. Our findings suggested that CAOS could be cost‐effective under several assumptions, such as the high surgical volume mentioned in almost all studies. The costs associated with the use of CAOS, ranging from US$400,000 (£290,000) to US$700,000 (£502,000), are a major concern, and the annual maintenance fees can reach £140,000 [66]. In the analysis models, a high number of surgical procedures could reduce the average cost per patient incurred by CAOS. In addition, the high volume could make the distinction of outcomes between the CAOS and conventional groups visible. Besides, the high volume helps surgeons overcome the learning curve. The annual cases suggested by these studies were over 250 for CAOS TKA and 100 for CAOS UKA. Our review seemed to reveal a dilemma for low‐volume institutes where the improved clinical outcomes from CAOS might not be equated with the increased economic investments in current settings. Although the employment of this new device can probably attract more patients, hospital administrators should balance the investments, volumes, and clinical outcomes. Alternatives other than purchase, such as renting or cost‐sharing strategies for low‐volume institutes, could also be considered to reduce the expenditures for employing CAOS.

Three CEAs about CAOS for spinal surgery were identified, and based on their results, the answer remains uncertain. The studies by Passias et al. [25] and Garcia et al. [55] showed completely contradictory results, even in the same procedure, which is MIS lumbar interbody fusion. We believed that the contradiction was due to the difference between their economic evaluation methods. In the study by Passias et al. [25], the purchase costs of CAOS were integrated and allocated to the cost per patient based on surgical volume, whereas in the study by Garcia et al. [55], the purchase costs of CAOS were not taken into account, resulting in a sharp decrease in the total costs of CAOS procedure. This might explain why the results of Garcia et al. [67] were positive and the results of Passias et al. [25] were negative. Additionally, a previous study demonstrated that CAOS might fail to reduce the postoperative complications in spinal surgery [67], which also have a negative impact on health and economics. With the increasing application of CAOS in spinal surgery, further studies are needed to explore their cost‐effectiveness and facilitate resource allocation decisions.

One of the most important goals of CAOS is to achieve a lower failure rate in surgery. In our review, CAOS arthroplasty demonstrated a longer survival than manual operation in the simulation models [43, 47, 51, 53]. As for spinal surgery, CAOS could also increase the accuracy of pedicle screw insertion and reduce the risk of revision [56]. Most of these studies attributed the better longevity of CAOS to the ability of CAOS that could obtain a better alignment of implants. The revision rate of CAOS also significantly influenced the cost‐effectiveness. Moschetti et al. [48] stated that the 2‐year revision rate should be lower than 1.2% to achieve a cost‐effective CAOS UKA [48]. Burn et al. [68] studied the cost‐effectiveness of CAOS TKA and THA at a given WTP threshold of £20,000 to £30,000 per QALY [68]. In their analysis, the revision rate was set at 5.3% for CAOS TKA and 8.2% for CAOS THA. The authors found that a 50% relative reduction in revision rate could improve the threshold with £1094 for knee replacement and £1347 for hip replacement [68]. In the CEAs for CAOS, especially when using economic models, researchers should carefully estimate the revision rate of CAOS as the economic models are extremely sensitive to these values. There is also a call for studies to improve the survival of computer‐assisted surgeries.

The majority of the studies involved were of high quality, but there were also weaknesses. Most studies used data from registries or publications. Only one study employed prospective data [56], which is considered the best available source [42]. Future studies on CEAs of CAOS would benefit from an RCT design and data collection. The potential factors related to the quality of CEAs defined in previous studies were also evaluated in this review, but none of them showed a statistical relationship. It should be noted that over half of the CEAs for CAOS in this review were funded by industries or conducted by authors who had a conflict of interest, although we are not inferring that these studies are biased.

Currently, published CEAs for CAOS are limited to the disciplines of primary joint arthroplasty and spinal surgery. However, CAOS has been used in complex revision arthroplasty [69], traumatology [70], and bone tumor surgery [71]. MacAskill et al. [69] and Zhang et al. [72] reported two cases of revision TKA and a case of revision THA using the MAKO robotic system (Stryker). Several studies employed the navigation system to facilitate internal fixation in patients with hip fractures [70, 73, 74]. However, no CEA has evaluated their economic outcomes. Thus, further studies are needed in these areas.

We noted several limitations in this review. As this is a systematic review, it may be unavoidable that relevant studies are missing. The paucity of available studies and heterogeneity of the reported outcomes limited the conclusions. However, we provided an overview of CAOS where there are few CEAs, so future studies are needed. We also used QHES to evaluate the quality of the articles. Although this scale has been employed for evaluation in many economic analyses, its primary focus is the quality of reporting [31]. The assessment of the relationship between quality and associated factors should also be interpreted with caution.

## 5 CONCLUSION

There are a small number of CEAs of CAOS, and our current review of available studies found that CAOS in joint arthroplasty may be cost‐effective under various assumptions, particularly in the case of high surgical volumes. Its cost‐effectiveness in spinal surgery needs more research effort to be clearly presented. Although current CEAs are of high quality, requiring more attention to their methodology. Further studies are needed to assess the cost‐effectiveness of CAOS in different areas of orthopedics.

## AUTHOR CONTRIBUTIONS

**Hua Li**: Data curation (equal); formal analysis (equal); methodology (equal); writing–original draft (lead); writing–review and editing (equal). **Tengfeng Zhuang**: Methodology (equal); writing–original draft (equal); writing–review and editing (equal). **Wenrui Wu**: Formal analysis (supporting); investigation (equal); methodology (equal); resources (equal); writing–original draft (supporting); writing–review and editing (supporting). **Wenyi Gan**: Formal analysis (supporting); methodology (equal); resources (supporting); writing–original draft (supporting); writing–review and editing (supporting). **Chongjie Wu**: Formal analysis (supporting); writing–original draft (supporting); writing–review and editing (supporting). **Sijun Peng**: Methodology (supporting); resources (supporting); writing–original draft (supporting); writing–review and editing (supporting). **Songwei Huan**: Conceptualization (equal); methodology (equal); writing–original draft (equal); writing–review and editing (equal). **Ning Liu**: Conceptualization (lead); supervision (lead); writing–review and editing (lead).

## CONFLICT OF INTEREST

The authors declare no conflict of interest.

## ETHICS STATEMENT

This is a systematic review and no ethical approval is required.

## INFORMED CONSENT

Not applicable.

## Data Availability

Data sharing is not applicable to this article as no data sets were generated or analyzed during the current study.
